# Hyper-connectivity between the left motor cortex and prefrontal cortex is associated with the severity of dysfunction of the descending pain modulatory system in fibromyalgia

**DOI:** 10.1371/journal.pone.0247629

**Published:** 2022-05-27

**Authors:** Álvaro de Oliveira Franco, Camila Fernanda da Silveira Alves, Paul Vicuña, Janete Bandeira, Maria Adelia de Aratanha, Iraci L. S. Torres, Felipe Fregni, Wolnei Caumo

**Affiliations:** 1 Laboratory of Pain and Neuromodulation, Hospital de Clínicas de Porto Alegre, Porto Alegre, RS, Brazil; 2 Postgraduate Program in Medical Sciences, School of Medicine, Universidade Federal do Rio Grande do Sul, Porto Alegre, RS, Brazil; 3 Hospital Israelita Albert Einstein, São Paulo, SP, Brazil; 4 Translational Nucleus: Pain Pharmacology and Neuromodulation, Hospital de Clínicas de Porto Alegre, Porto Alegre, RS, Brazil; 5 Laboratory of Neuromodulation and Center for Clinical Research Learning, Physics and Rehabilitation Department, Spaulding Rehabilitation Hospital, Boston, MA, United States of America; 6 Pain and Palliative Care Service, Hospital de Clínicas de Porto Alegre, Porto Alegre, RS, Brazil; 7 Department of Surgery, School of Medicine, Universidade Federal do Rio Grande do Sul, Porto Alegre, RS, Brazil; Tokai University, JAPAN

## Abstract

**Introduction:**

The association between descending pain modulatory system (DPMS) dysfunction and fibromyalgia has been previously described, but more studies are required on its relationship with aberrant functional connectivity (FC) between the motor and prefrontal cortices.

**Objectives:**

The objective of this cross-sectional observational study was to compare the intra- and interhemispheric FC between the bilateral motor and prefrontal cortices in women with fibromyalgia, comparing responders and nonresponders to the conditioned pain modulation (CPM) test.

**Methods:**

A cross-sectional sample of 37 women (23 responders and 14 nonresponders to the CPM test) with fibromyalgia diagnosed according to the American College of Rheumatology criteria underwent a standardized clinical assessment and an FC analysis using functional near-infrared spectroscopy. DPMS function was inferred through responses to the CPM test, which were induced by hand immersion in cold water (0–1°C). A multivariate analysis of covariance for main effects between responders and nonresponders was conducted using the diagnosis of multiple psychiatric disorders and the use of opioid and nonopioid analgesics as covariates. In addition, we analyzed the interaction between the CPM test response and the presence of multiple psychiatric diagnoses.

**Results:**

Nonresponders showed increased FC between the left motor cortex (lMC) and the left prefrontal cortex (lPFC) (t = −2.476, p = 0.01) and right prefrontal cortex (rPFC) (t = −2.363, p = 0.02), even when both were considered as covariates in the regression analysis (lMC–lPFC: β = −0.127, t = −2.425, p = 0.021; lMC–rPFC: β = −0.122, t = −2.222, p = 0.033). Regarding main effects, a significant difference was only observed for lMC–lPFC (p = 0.035). A significant interaction was observed between the psychiatric disorders and nonresponse to the CPM test in lMC−lPFC (β = −0.222, t = −2.275, p = 0.03) and lMC−rPFC (β = −0.211, t = −2.2, p = 0.035). Additionally, a significant interaction was observed between the CPM test and FC in these two region-of-interest combinations, despite the psychiatric diagnoses (lMC−lPFC: β = −0.516, t = −2.447, p = 0.02; lMC−rPFC: β = −0.582, t = −2.805, p = 0.008).

**Conclusions:**

Higher FC between the lMC and the bilateral PFC may be a neural marker of DPMS dysfunction in women with fibromyalgia, although its interplay with psychiatric diagnoses also seems to influence this association.

## Introduction

Fibromyalgia (FM) is characterized by widespread pain and the coexistence of depressed mood, sleep disorders, and cognitive symptoms [1, 2]. Its worldwide prevalence ranges from 0.4% to 9.3% in the general population [3]; it is diagnosed in a ratio of approximately three women to one man and has an average age of onset between 30 and 50 years [4]. FM produces a substantial socioeconomic burden due to disability, representing approximately USD 1 billion in hospital charges in the USA in 9 years [5]. Approximately 55.8% of patients with FM less than 65 years old cannot work because of health problems, compared with 5.5% in controls. Additionally, 50.5% of FM patients in this age group filed social security disability applications [6]. The pathophysiological process underlying FM is hypothesized to be a maladaptive function of the central [7] and peripheral nervous systems [8–10], with an active area of research on the proportion and degree to which each nervous system component contributes to FM emergence. Dysfunctional neuroinflammation [11] and abnormal neurotransmitters [12] are associated with maladaptive neuroplasticity. This process may generate an imbalance of facilitation and inhibition in pain processing pathways. In addition, they can explain the hyper-inhibition of cortical excitability measures observed in FM [13] and the gray matter loss observed in neuroimaging studies [14–16]. Thus, these changes on the functional and structural levels are likely factors that determine FM symptom emergence and maintenance, which may be reflected in abnormal functional connectivity (FC) across pain processing networks [17–19].

Previous research demonstrated that in FM, the spectrum of disability and symptom severity is proportional to the dysfunction of the descending pain modulatory system (DPMS) [20]. Additionally, studies have presented resting-state FC abnormalities across the cortical areas related to top-down modulation of DPMS [21, 22]. The function of DPMS may be assessed by the conditioned pain modulation test (CPM test) [23]. When a subgroup of patients with FM is assessed using the CPM test, they consistently present ineffective endogenous pain modulation [24–29]. DPMS is a set of neural networks that encompasses subcortical structures (e.g., the periaqueductal substance (PAG), rostral medial medulla, nucleus accumbens, mesolimbic reward circuit, and amygdala) as well as cortical areas (e.g., the motor cortex (MC), prefrontal cortex (PFC), primary (S1) and secondary somatosensory cortices (S2), and cingulate) [30, 31]. Among the DPMS structures, the PFC and the limbic system play a significant role in encoding pain’s emotional and contextual components [32–34]. Patients with FM displayed increased activation of the dorsolateral prefrontal cortex (DLPFC) following painful stimulation [35] and during anticipation of pain [36]. The MC is a key top-down modulatory effector that plays a pivotal role in pain outcomes [37]. Furthermore, the reversal of grey matter loss in the DLPFC and MC occurred following therapeutic interventions in patients with chronic pain [38–41]. Both the DLPFC and the MC already represent target areas for noninvasive top-down neuromodulatory intervention, such as transcranial magnetic stimulation (TMS) and transcranial direct current stimulation (tDCS) [35, 42–45]. Finally, FC can be assessed using functional near-infrared spectroscopy (fNIRS), an imaging method employed for cortical activity mapping. It has excellent temporal resolution that can detect changes in oxygenated hemoglobin (HbO) and deoxygenated hemoglobin levels because of the transient dynamics of neurovascular coupling elicited by neuronal activation, a phenomenon that constitutes the hemodynamic response [35, 46–48].

This study was conducted to test whether the FC between the bilateral MC and PFC measured using fNIRS could discriminate between women with FM according to the spectrum of responders and nonresponders to the CPM test. The paradigm of the CPM test was evaluated by the change in the numerical pain scale (NPS) (score ranging from 0 to 10). Quantitative sensory testing (QST) was performed concurrently with the heterotopic nociceptive stimulus at the same temperature at which patients reported an NPS score of 6/10 during the QST without heterotopic stimulus. The reported NPS scores were compared before and after the heterotopic nociceptive stimulus, which was induced by immersion of the dominant hand in cold water (0–1°C) for 15 seconds. It was hypothesized that the FC between these cortical areas involved in top-down modulation may be a biomarker of DPMS dysfunction in FM.

## 2. Materials and methods

### 2.1. Design overview, settings, and participants

This study’s protocol was approved by the Institutional Review Board (IRB) of the Hospital de Clínicas de Porto Alegre (HCPA), Brazil, and registered in the Certificate of Presentation of Ethical Appreciation (CAAE registry n^o^ 2017–0329). Written informed consent was obtained from all participants before their inclusion. For this cross-sectional observational study, the study enrollment period ranged from January 2018 to December 2019.

### 2.2. Recruitment and inclusion and exclusion criteria

Forty-three right-handed literate females aged 30 to 65 years and diagnosed with FM were enrolled. They were seen in the outpatients’ chronic pain wards of the HCPA, Porto Alegre, Brazil, and digital media publicity. The diagnosis of FM was made according to the American College of Rheumatology 2016 [49] and confirmed by physicians with more than ten years of experience in chronic pain care. The criteria set defines fibromyalgia in adults through the presence of the following four criteria: (1) Generalized pain, in 4 of 5 regions. (2) Widespread pain index (WPI) > 7 and symptom severity scale (SSS) >5, OR WPI 3–6 and SSS >9. (3) Symptoms present at a similar level for at least 3 months. (4) The diagnosis of fibromyalgia is irrespective of other diagnoses [49].

This study included only women due to the higher prevalence of FM in women than men [4]. Patients were considered eligible if they had a clinically significant level of pain in the last 3 months–defined as a score greater than or equal to six points (i.e., moderate or severe pain) in NPS [8, 50]. All patients were fluent in Portuguese. Patients were excluded if they had rheumatoid arthritis, systemic lupus erythematosus, or another autoimmune, neurologic, or oncologic disease. Those with current uncompensated clinical disease (e.g., ischemic heart disease, chronic kidney disease, or hepatic disease) or using cannabis or recreational psychotropic drugs in the last six months were similarly excluded.

### 2.3. Sample size estimation

A lack of previous knowledge about brain FC according to the DPMS in FM prevented defining an a priori sample. Thus, a post-hoc power analysis calculation was performed and yielded an estimated sample of 37 patients ensuring detection of an effect size (f2) of 0.25 during multiple regression analysis, allowing three predictors based on the consideration of type I and II errors 0.05 and 0.20, respectively [51].

### 2.4. Dependent and independent variables of primary interest

Ipsi- and contralateral FC between the MC and PFC were the dependent variables. The main factor of interest was the function of DPMS evaluated by the change in the NPS score during the CPM-test.

### 2.5. Instruments and assessments

#### 2.5.1. Functional near-infrared spectroscopy assessment

**Fig 1:**
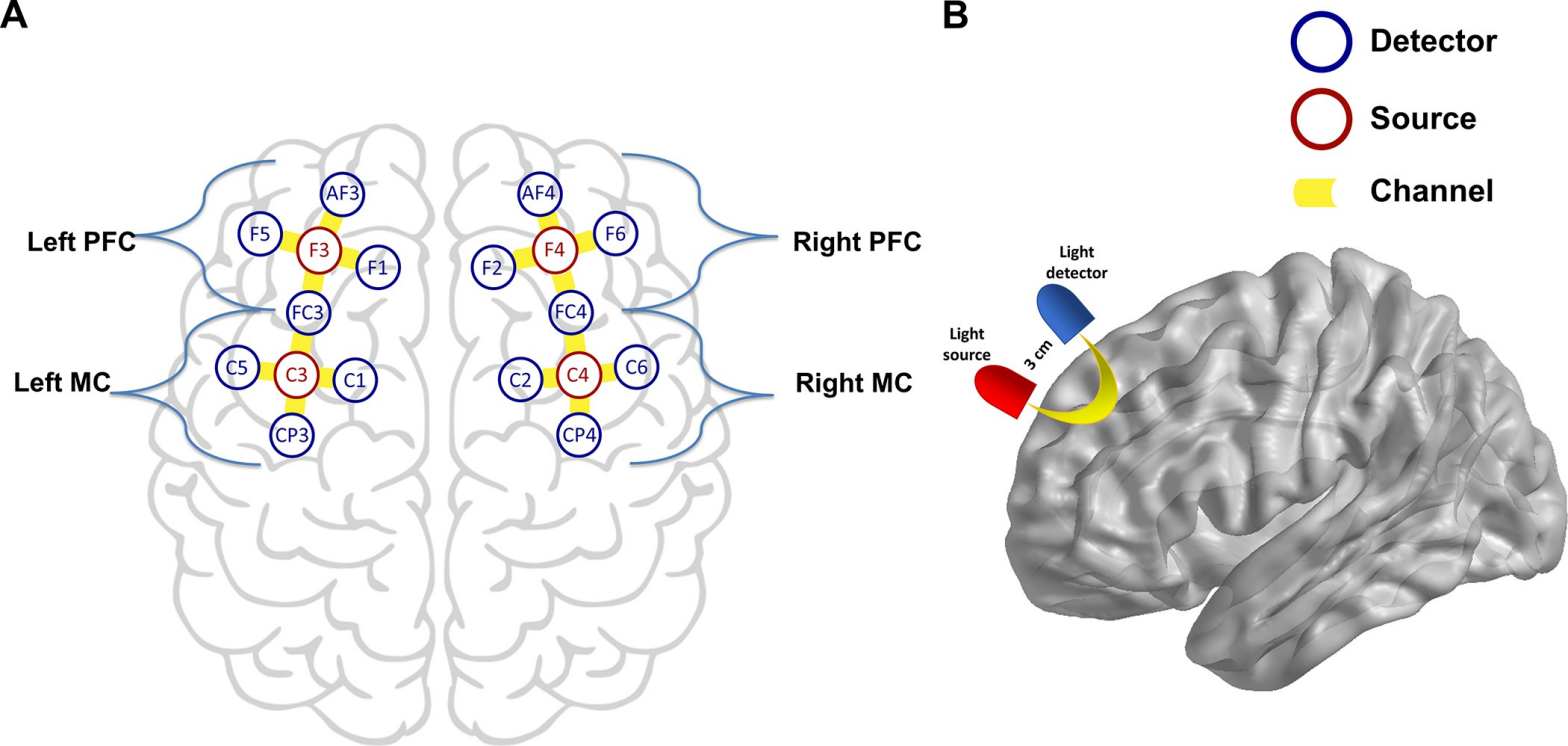
fNIRS montage and representation of one channel. **(A)** Sources and detectors were arrayed in a 10–10 system. (**B)** Illustrative demonstration of near-infrared diffuse reflection path between source and detector.

Cortical activation was evaluated by fNIRS using a NIRx® continuous waveform NirScout® near-infrared spectroscopy device (NIRx Medical Technologies, Glen Head, NY, USA) with a scan rate of 15 Hz and dual-wavelength light-emitting diode sources (760 and 850 nm). Four sources and 14 detectors spaced about 3 cm apart were placed over the scalp using caps provided by EasyCAP®, with 16 channels in total (Fig 1). Probe localization was established using the international 10–10 electroencephalography system. In the used montage, sources were placed in the F3, F4, C3, and C4 locations and detectors in the AF3, F5, FC3, F1, C5, CP3, C1, AF4, F6, FC4, F2, C6, CP4, and C2 locations. Four regions of interest (ROI) were defined: left PFC (source: F3; detectors: AF3, F1, FC3, F5), left MC (source: C3; detectors: FC3, C1, CP3, C5), right PFC (source: F4; detectors: AF4, F6, F2, FC4), right MC (source: C4; detectors: FC4, C6, CP4, C2) (Fig 1A). Recordings were made using the NIRStar® version 14.2 software program (NIRx Medical Technologies, Glen Head, NY, USA) and were compiled during a single session for each patient.

**Fig 2:**
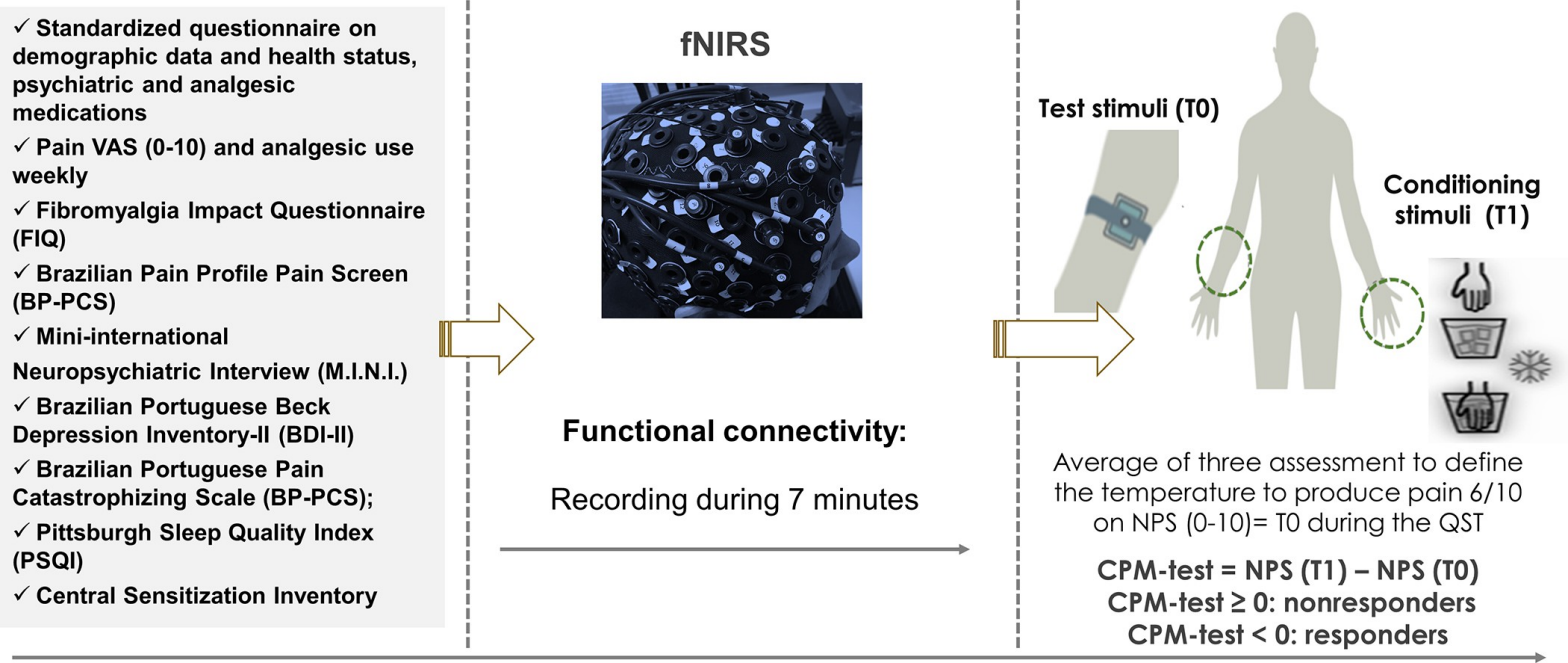
Study flow diagram. Timeline assessments. CPM-test: condition pain modulation test; fNIRS: functional near-infrared spectroscopy. Pain VAS: Pain visual-analog scale. NPS: numeric pain scale. QST: Quantitative Sensory Testing. T0: mean temperature (three measurements per individual) at which the subjects reported a pain score of 6/10 on the NPS (0–10) during the QST before the heterotopic stimulus. T1: temperature equivalent to T0 but applied during hand water cold immersion. NPS(T0): NPS pain score during application of T0. NPS(T1): NPS pain score during application of T1.

After adjusting the NIRS cap in the scalp, a black cover was placed over it to reduce the degree of environmental light disturbance. Source-detection calibration and recording-checked signal quality were only confirmed if the calibration returned an assessment of “excellent” quality in at least 14 out of 16 channels and if the other two channels were at least “acceptable” on a qualitative scale based on gain, amplitude, coefficient of variation of noise, and dark noise. Patients were instructed to sit on a comfortable chair and to maintain a still position. They were asked to fixate their gaze on a black cross fixed on the front wall at eye level 1.5 meters ahead of the chair. Lastly, they were asked to try to think of nothing. Recording of fNIRS activity was performed for seven minutes, which was the time length used in the FC analysis (Fig 2).

#### 2.5.2. Functional connectivity analysis

The seven-minute recorded data of each subject were preprocessed using the Brain AnalyzIR® [47] software on the MATLAB (The MathWorks, Inc., Natick, MA, USA)) platform. For the assessment of functional connectivity values, raw data were downsampled to 1 Hz to adequately address the high level of temporal autocorrelation in fNIRS signals. They were subsequently converted to optical density and then to oxyhemoglobin concentration variation (HbO) using the Beer-Lambert law’s modified version [52].

Data were treated through an autoregressive prewhitening model to correct structured noisy and serially correlated error effects (e.g., physiological noise) together with iterative reweighting through a robust regression method addressing outliers (e.g., motion artifacts) with no use of any band/high/low filtering [53, 54]. This method is appropriate for the necessary treatment of serially correlated errors, colored noise, and motion artifacts, dispensing other forms of data processing [53, 55]. In this study, the HbO signal was included in the analysis based on previous reports, which have indicated that this is the most sensitive variable by which to estimate cortical activity via inferences concerning neurovascular coupling based on hemodynamic response [30, 56–58]. Finally, Pearson correlation values were computed for all possible pairs of channels across the time series, and the resulting sample correlation coefficients underwent a Fisher Z-transformation. The Z-values were then averaged for each ROI in each subject (four ROIs × 37 subjects) [59].

#### 2.5.3. Conditioned pain modulation test

A thermode (30 × 30 mm) was attached to the skin on the mid-forearm ventral aspect, as classically performed for CPM evaluation, having been already compared with other anatomical sites in another study [60]. Testing stimulus using the thermode was applied in the non-dominant side whereas the conditioning stimulus by a cold pressor test was applied in the dominant side. Since all subjects were right-handed, the thermode was attached in the left arm across all stages. The thermode was a standardized piece of equipment. The CPM-test assessment encompassed three stages: (**i**) to perform Quantitative Sensory Testing (QST), the temperature of the thermode was set at 32°C and then it was heated at a rate of 1°C per second up to 52°C, when the temperature was set to drop again thereafter. Subjects were asked to report when the temperature reached a level of pain consistent with an NPS score of six points in ten (i.e., “6/10 score”). This assessment was replicated three times and averaged the temperatures to determine the necessary value to achieve a NPS 6/10 score (T0). All three replicates were performed with an interstimulus interval of 40 seconds, and the position of the thermode was slightly altered between trials. (**ii**) Five minutes after assessing stage I, patients immersed their right hands up to their wrists into the water at a temperature of between 0°C and 1°C for 15 seconds. After that, the QST was reapplied with the thermode using the stimulation area of stage I. **(iii)** The CPM-test score was calculated according to the change in the NPS score retrieved after applying a new QST with the temperature set as the necessary value for a NPS 6/10 score according to the initial trial (T0), while patients maintained their right hands in cold water immersion (QST + CPM-test). The CPM-test absolute score is a difference between the NPS-score after and before the cold stimulus. Patients were considered responders to the CPM-test if their difference value was less than zero (i.e., the NPS-score after cold stimulus reflected pain inhibition in relation to the original NPS-score for the same testing stimulus magnitude), and nonresponders if the difference value was greater than or equal to zero [61]. It is essential to realize that the CPM-test’s negative values suggest a higher effect of heterotopic stimulus inhibiting the test stimulus (i.e., “pain inhibits pain”)—in other words, a better function of the DPMS.

#### 2.5.4. Pain measures, psychological assessments, sleep quality, and sociodemographic characteristics

The Fibromyalgia Impact Questionnaire (FIQ) [62] was used to evaluate the impact of life quality symptoms. Pain Visual Analogue Scale (VAS) was used for rating the subjective pain intensity in most days of the last three months through a ruler ranging from 0 mm to 100 mm in which subjects were asked to indicate their pain level, with 0 mm being no pain at all and 100 mm the worst pain in life. The Pain Catastrophizing Scale (PCS) [63] was used to assess the identification of an individual’s pain catastrophizing into three dimensions: rumination, magnification, and helplessness. Beck Depression Inventory-II [64] was used to evaluate depressive symptoms, and Central Sensitization Inventory [65] was used to measure symptoms related to central sensitization syndrome. Over the last month, sleep quality and sleep patterns were evaluated by the Pittsburgh Sleep Quality Index (PSIQI) [66].

A standardized query was used to assess demographic data and medical comorbidities, which included information about age, sex, years of education, and lifestyle habits. Patients also provided information about their health status, including clinical diagnoses. Diagnosis of psychiatric disorders was established through the Mini International Neuropsychiatric Interview (MINI) [67]. A considerable number of patients displayed more than one psychiatric diagnosis, with almost all presenting at least one. For the analysis, subjects diagnosed with more than one psychiatric disorder according to the MINI were considered to have multiple psychiatric diagnoses. A specific questionnaire evaluated all medications used and their daily doses (e.g., antidepressants, antiepileptic, benzodiazepines, nonopioid and opioid analgesics, etc.). The opioid use was calculated by mean morphine-equivalent dose (MED) per day [68]. They were classified with minimal opioid or no use if the mean daily MED was less than 5 mg or if self-reported opioid use was less than twice a week in the previous 28 days. If they self-reported a mean daily MED equal to or greater than 5 mg for most days for the last three months, they were classified as regular opioid users. Nonopioid analgesic use was defined by analgesic use per week on most days of the previous month. For data analysis, the analgesic use was dichotomized into those who used analgesics less than four days per week or used equal or higher than four days per week. This approach was adopted because chronic pain patients can change their rescue analgesic use each week depending on pain levels.

Among the efforts to reduce potential sources of bias, all assessments were conducted by researchers with vast clinical expertise to treat outpatients in a pain clinic, and the diagnoses were done according to the pre-specified criteria described above. Two evaluators with specific training were responsible for all assessments and for applying the standardized protocol to assess the CPM-test and the evaluation using the fNIRS. All patients and evaluators were fluent in Portuguese. All questionnaires were validated in Portuguese.

### 2.6. Statistical analysis

Pearson’s chi-squared test, Fisher’s exact tests, and t-tests for independent samples were used to compare categorical and continuous variables between groups. The Shapiro-Wilk test was used to test for normality. The FC Z-values among four ROIs (ROIs: left MC, right MC; left PFC, right PFC) taken in pairs (ROI−ROI) were compared by t-test for independent samples in univariate analysis. A multivariate covariance analysis (MANCOVA) model was used to compare ROI−ROI FC Z-values between responders and nonresponders to CPM-test adjusted by the following factors: opioid analgesic use (Minimal/no use, or regular opioid use), nonopioid analgesic use, and multiple psychiatric disorders (>1 according to the MINI). To identify the source of significant differences was used Bonferroni’s Multiple Comparison Test. Statistical significance was set to a p-value of 0.05, 2-tailed. The statistical analysis was conducted in IBM SPSS Statistics for Windows, version 22.0 (IBM Corp., Armonk, N.Y., USA).

## 3. Results

### 3.1. Patient characteristics

A total of 43 subjects were enrolled, of which six were excluded, three for missing data and three due to poor quality of data from fNIRS measures. The final sample was composed of 37 participants (23 responders and 14 nonresponders to the CPM-test). The characteristics of the sample and comparative analyses between responders and nonresponders are presented in Table 1. There were no statistical differences between responders and nonresponders in sociodemographic characteristics, psychological measures, sleep quality, psychotropic medications, and analgesic use. However, nonresponders, compared to responders, presented higher scores in the Visual Analogue Scale in the last three months with a borderline significance.

**Table 1:** Demographic and clinical characteristics of the study sample (n = 37).

	Responders (n = 23)	Nonresponders (n = 14)	p-value
Age (years)	49.304 (9.693)	51.071 (9.988)	0.598
Body index	28.907 (4.417)	30.009 (4.99)	0.511
Education (years)	13.174 (4.206)	10.857 (4.849)	0.134
Smoking (*Yes*/*No*)	6 (26.1%)/17	3 (21.4%)/11	
Clinical Comorbidity (*Yes*/*No)*	15 (65.2%)/8	9 (64.3%)/5	
Hypertension (*Yes*)	9 (39.1%)	7 (50%)	
Diabetes (*Yes*)	3 (13%)	1 (7.1%)	
Thyroid disease (*Yes*)	4 (17.4%)	5(35.7%)	
Asthma (*Yes*)	7 (30.4%)	1 (7.1%)	
**Pain, sleep quality, and psychological measures**			
Beck Depression Inventory	25.304 (12.542)	25.5 (10.544)	0.961
Central Sensitization Inventory	68.522 (11.812)	68.357 (14.008)	0.970
Pain Catastrophizing Scale	34.565 (11.057)	35.429 (13.715)	0.835
Fibromyalgia Questionnaire M2	70.463 (12.689)	72.488 (19.008)	0.708
Pittsburgh Sleep Quality Index	12.913 (3.907)	12.643 (4.03)	0.841
American College of Rheumatology—2016			
Revisions to the 2010/2011 Fibromyalgia Diagnostic Criteria	23.87 (2.928)	23.071 (4.159)	0.498
Visual Analogue Scale (0–10) of pain in the last 3 months	8.304 (1.324)	9.128 (0.921)	0.05
Nonopioid analgesic use in the last month ≥4 days per week (*Yes*)*	5 (21.7%)	4 (28.6%)	
Dipyrone (*Yes*)	5 (21.7%)	4 (28.6%)	
Paracetamol (*Yes*)	7 (30.4%)	4(28.6%)	
Dorflex® (*Yes*)	11 (47.8%)	6 (42.9%)	
Cyclobenzaprine (*Yes*)	12 (52.2%)	1 (7.1%)	
NSAID (*Yes*)	22 (95.7%)	13 (92.9%)	
Use opioid analgesic use			
Minimal or no use	18 (78.3%)	12 (85.7%)	
Regular opioid use	5 (21.7%)	2 (14.3%)	
Psychiatric disorder according to the MINI (*Yes*/*No*) **	18 (78.3%)/5	13 (92.9%)/1	
Major Depressive Disorder	14 (60.9%)	13 (92.9%)	
Generalized Anxiety Disorder	10 (43.5%)	3 (21.4%)	
Panic Disorder	4 (17.4%)	2 (14.3%)	
Current use of neuropsychiatric medication (*Yes*/*No*) *	13 (72.2%)/5	11 (84.6%)	
Tricyclic antidepressants (yes)	2 (8.7%)	5 (35.7%)	
Dual-action antidepressants *(Yes)*	11 (47.8%)	4 (28.6%)	
Selective serotonin reuptake inhibitors (*Yes*)	5 (21.7%)	4 (28.6%)	
Antipsychotic (*Yes*)	2 (8.7%)	1 (7.1%)	
Antiepileptic (*Yes*)	8 (34.8%)	4 (28.6%)	
Benzodiazepines (*Yes*)	3 (13%)	4 (28.6%)	

Values are given as the mean (SD) or frequency.

NSAID: nonsteroidal anti-inflammatory drugs; MINI: Mini International Neuropsychiatric Interview.

*Some patients used more than one type of drug

**Some patients had more than one psychiatric disorder. Dorflex® (300 mg of dipyrone monohydrate, 35 mg of orphenadrine citrate, and 50 mg of anhydrous caffeine).

### 3.2. Univariate analysis according to the spectrum of responders and nonresponders to CPM-test in the FC

According to the spectrum to responders and nonresponders to CPM-test, the mean (standard deviation) of cortical FC is presented in Table 2. Nonresponders to the CPM-test showed higher FC between the left MC and the left PFC (t = -2.476, p = 0.01) compared to responders. Also, nonresponders presented higher FC levels between the left MC and the right PFC (t = -2.363, p = 0.02).

**Table 2:** Relationship of the outcomes and the main interest factor according to the spectrum of responders and nonresponders to CPM-test (n = 37).

Region of interest connectivity	Responders	Nonresponders	t	p-value
Left prefrontal cortex–Left motor cortex	0.607 (0.165)	0.738 (0.138)	-2.476	0.01
Left prefrontal cortex–Right prefrontal cortex	0.728 (0.189)	0.778 (0.133)	-0.861	0.39
Left prefrontal cortex–Right motor cortex	0.521 (0.163)	0.608 (0.085)	-1.831	0.07
Left motor cortex–Right prefrontal cortex	0.524 (0.165)	0.648 (0.131)	-2.363	0.02
Left motor cortex–Right motor cortex	0.518 (0.224)	0.610 (0.149)	-1.370	0.17
Right prefrontal cortex–Right motor cortex	0.544 (0.199)	0.617 (0.133)	-1.214	0.23

Data are presented as the mean (standard deviation) (n = 37).

### 3.3. Multivariate analysis of the relationship between the FC according to the spectrum of responders and nonresponders to CPM test

The results of the MANCOVA model analysis for FC with CPM-test as the dependent variable are presented in Table 3. FC between MC and PFC was compared between responders and nonresponders to the CPM-test. The presence of multiple psychiatric disorders, number of nonopioid analgesics daily used, and the number of opioid medications were used as independent variables. The MANCOVA model using Bonferroni’s Multiple Comparison Test revealed a significant relationship between the responders and nonresponders groups and the outcomes related to FC (Hotelling’s Trace = 1.21, F (6) = 5.47, P = 0.001). This analysis presented a power of 0.98.

**Table 3:** MANCOVA analysis of the relationship between the FC according to the spectrum of responders or nonresponders according to change in NPS (0–10) during the CPM-test (n = 37).

**(A) Main effects**						
**Dependent Variable**	**Type III Sum of Squares**	**DF**	**Mean Square**	**F**	**Sig.**	**Partial Eta Squared**
Left prefrontal cortex–Left motor cortex	0.271	4	0.068	2.958	0.035	0.27
Left prefrontal cortex–Right prefrontal cortex	0.029	4	0.007	0.232	0.918	0.028
Left prefrontal cortex–Right motor cortex	0.119	4	0.03	1.501	0.225	0.158
Left motor cortex–Right prefrontal cortex	0.153	4	0.038	1.510	0.223	0.159
Left motor cortex–Right motor cortex	0.112	4	0.028	0.656	0.627	0.076
Right prefrontal cortex–Right motor cortex	0.174	4	0.044	1.423	0.249	0.151
**B. Coefficient**						
	**B**	**Std. Error**	**t**	**Sig.**	**CI 95%**	
**Left prefrontal cortex–Left motor cortex: Dependent Variable**						
*Intercept*	0.844	0.163	5.178	0.000	(0.512 to 1.176)	
Responders⁄ Nonresponders	-0.127	0.052	-2.425	0.021	(-0.234 to -0.020)	
Multiple psychiatric disorders according to the MINI	-0.066	0.056	-1.181	0.246	(-0.181 to 0.048)	
Regular or higher dose of opioids	0.092	0.066	1.396	0.172	(-0.042 to 0.225)	
Nonopioid analgesic use in the last month ≥4 days per week	-0.091	0.061	-1.506	0.142	(-0.214 to 0.032)	
**Left prefrontal cortex–Right prefrontal cortex: Dependent Variable**						
*Intercept*	0.813	0.192	4.245	0.000	(0.423 to 1.203)	
Responders⁄ Nonresponders	-0.053	0.062	-0.858	0.398	(-0.178 to 0.073)	
Multiple psychiatric disorders according to the MINI	-0.031	0.066	-0.470	0.642	(-0.166 to 0.104)	
Regular opioid use	0.013	0.077	0.175	0.862	(-0.144 to 0.171)	
Nonopioid analgesic use in the last month ≥4 days per week	-0.003	0.071	-0.036	0.971	(-0.147 to 0.142)	
**Left prefrontal cortex–Right motor cortex: Dependent Variable**						
*Intercept*	0.592	0.151	3.913	0.000	(0.284 to 0.9)	
Responders⁄ Nonresponders	-0.075	0.049	-1.549	0.131	(-0.174 to 0.024)	
Multiple psychiatric disorders according to the MINI	0.007	0.052	0.140	0.890	(-0.099 to 0.114)	
Regular opioid use	0.072	0.061	1.175	0.249	(-0.053 to 0.196)	
Nonopioid analgesic use in the last month ≥4 days per week	-0.076	0.056	-1.350	0.186	(-0.19 to 0.039)	
**Left motor cortex–Right prefrontal cortex: Dependent Variable**						
*Intercept*	0.667	0.171	3.894	0.000	(0.318 to 1.016)	
Responders⁄ Nonresponders	-0.122	0.055	-2.222	0.033	(-0.235 to -0.01)	
Multiple psychiatric disorders according to the MINI	-0.031	0.059	-0.525	0.603	(-0.152 to 0.09)	
Regular or higher dose of opioids	0.045	0.069	0.649	0.521	(-0.096 to 0.185)	
Nonopioid analgesic use in the last month ≥4 days per week	-0.027	0.064	-0.428	0.672	(-0.157 to 0.102)	
**Left motor cortex–Right motor cortex: Dependent Variable**						
*Intercept*	0.546	0.222	2.458	0.02	(0.093 to 0.999)	
Responders⁄ Nonresponders	-0.083	0.071	-1.166	0.252	(-0.229 to 0.062)	
Multiple psychiatric disorders according to the MINI	0.006	0.077	0.084	0.933	(-0.150 to 0.163)	
Regular opioid use	0.073	0.089	0.818	0.419	(-0.109 to 0.255)	
Nonopioid analgesic use in the last month ≥4 days per week	-0.048	0.083	-0.585	0.563	(-0.216 to 0.120)	
**Right prefrontal cortex–Right motor cortex: Dependent Variable**						
*Intercept*	0.473	0.188	2.510	0.017	(0.089 to 0.856)	
Responders⁄ Nonresponders	-0.050	0.061	-0.82	0.418	(-0.173 to 0.074)	
Multiple psychiatric disorders according to the MINI	0.067	0.065	1.026	0.312	(-0.066 to 0.199)	
Regular opioid use	0.105	0.076	1.386	0.175	(-0.049 to 0.26)	
Nonopioid analgesic use in the last month ≥4 days per week	-0.099	0.07	-1.417	0.166	(-0.242 to 0.043)	

MINI: Mini International Neuropsychiatric Interview.

DF: degrees of freedom.

^*a*^ *R*^*2*^ *adjusted = 0*.*179*

^*b*^ *R*^*2*^ *adjusted = 0*.*093*

^*c*^ *R*^*2*^ *adjusted = 0*.*053*

^*d*^ *R*^*2*^ *adjusted = 0*.*054*

^*e*^ *R*^*2*^ *adjusted = 0*.*04*

^*f*^ *R*^*2*^ *adjusted = 0*.*045*.

In the regression analysis, nonresponders showed higher FC between the left MC and the left and right PFC. No effect was observed between the left PFC−right PFC, left PFC−right MC, left MC−right MC, or right PFC−right MC. Adjusted R-squared of FC presented low values for all ROI−ROI pairs except for the left MC−left PFC (R^2^ adjusted = 0.179).

**Fig 3:**
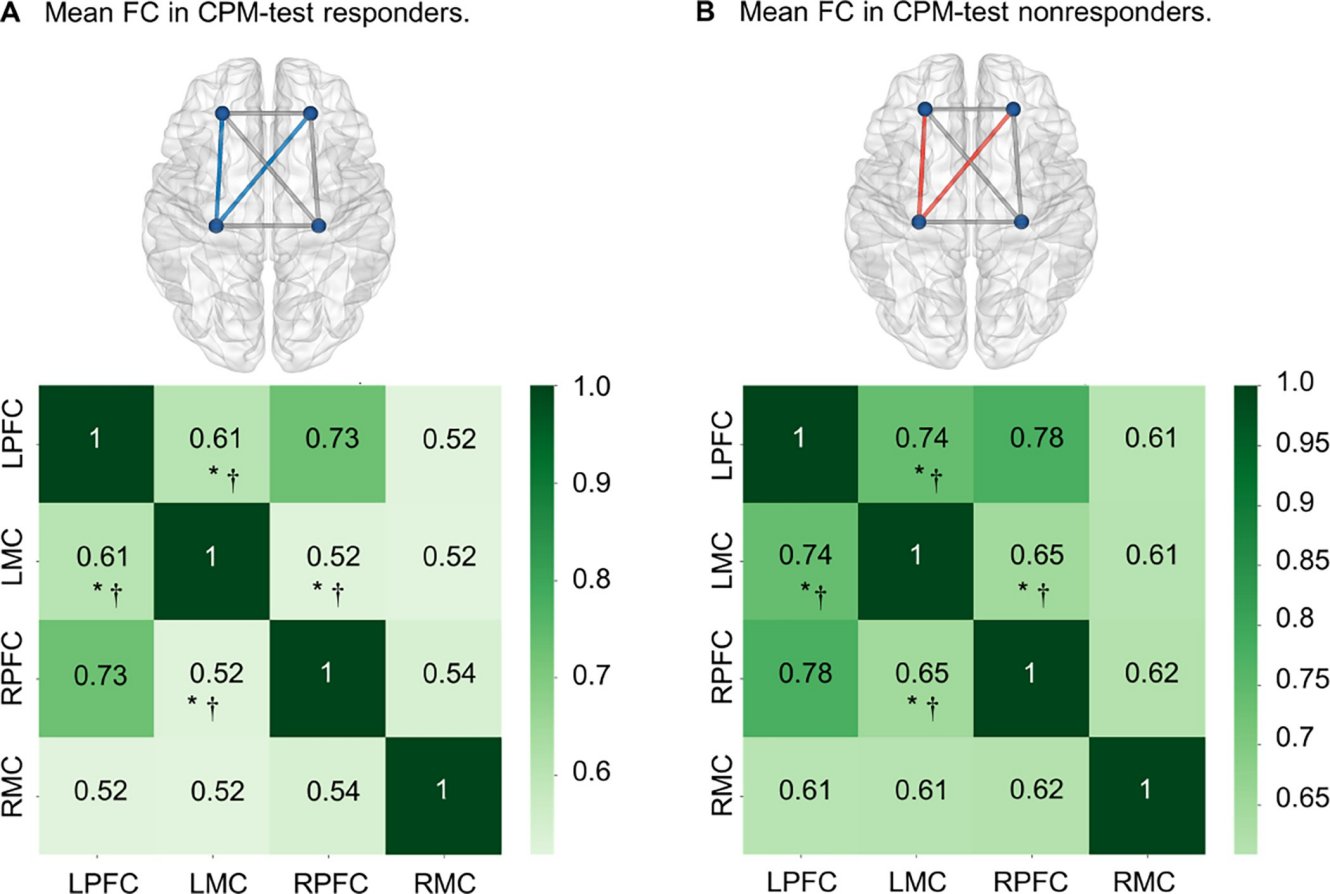
Heatmap visualization of FC across regions of interest in Z-values. Nonresponders presented higher connectivity than responders between LMC and bilateral PFC. Blue color edges represent significantly lower values and red color edges represent significantly higher values, relative to the comparison between groups. **A.** Mean FC by region of interest in CPM-test responders. **B.** Mean FC by region of interest in CPM-test nonresponders. FC: functional connectivity; CPM-test: conditioned pain modulation test. LPFC: Left prefrontal cortex; LMC: Left motor cortex; RPFC: Right prefrontal cortex; RMC: Right motor cortex. *p<0.05. † FC between regions of interest that were associated with CPM-test groups despite the interaction effect of multiple psychiatric diagnoses.

Psychiatric disorders, regular opioid, and nonopioid analgesic use, when taken as regressors, were not associated with any ROI−ROI FC. The heatmap visualization of FC across regions of interest in Z-values is shown in Fig 3.

### 3.4. Multivariate analysis to examine the FC considering the interaction between psychiatric disorders with the spectrum of responders and nonresponders to CPM test

In the analysis shown in Table 3, the presence of psychiatric disorders according to the MINI was a covariate not related to the FC. To further explore its effect in this relationship, a MANCOVA was conducted to examine the interaction effects of psychiatric disorders on the FC and CPM-test (Table 4).

**Table 4:** MANCOVA analysis of the relationship between the FC according to the interaction analysis between the spectrum of responders or nonresponders on the CPM-test by psychiatric disorders according to the MINI (n = 37).

**(A) Main effects**								
**Dependent Variable**	**Type III Sum of Squares**			**df**	**Mean Square**	**F**	**Sig.**	**Partial Eta Squared**
Left prefrontal cortex–Left motor cortex	0.266			3	0.089	3.957	0.016	0.265
Left prefrontal cortex–Right prefrontal cortex	0.061			3	0.02	0.688	0.566	0.059
Left prefrontal cortex–Right motor cortex	0.066			3	0.022	1.057	0.381	0.088
Left motor cortex–Right prefrontal cortex	0.25			3	0.083	3.856	0.018	0.26
Left motor cortex–Right motor cortex	0.107			3	0.036	0.857	0.473	0.072
Right prefrontal cortex–Right motor cortex	0.076			3	0.025	0.779	0.514	0.066
**Conditioned pain modulation**								
Left prefrontal cortex–Left motor cortex	0.134			1	0.134	5.987	0.02	0.154
Left prefrontal cortex–Right prefrontal cortex	0.046			1	0.046	1.544	0.223	0.045
Left prefrontal cortex–Right motor cortex	0.005			1	0.005	0.265	0.610	0.008
Left motor cortex–Right prefrontal cortex	0.17			1	0.17	7.868	0.008	0.193
Left motor cortex–Right motor cortex	0.056			1	0.056	1.352	0.253	0.039
Right prefrontal cortex–Right motor cortex	0.009			1	0.009	0.273	0.605	0.008
***Interaction analysis according to CPM-test categories classified in responders⁄ nonresponders***								
* Multiple psychiatric disorders according to the MINI								
Left prefrontal cortex–Left motor cortex	0.116			2	0.058	2.591	0.09	0.136
Left prefrontal cortex–Right prefrontal cortex	0.04			2	0.02	0.669	0.519	0.039
Left prefrontal cortex–Right motor cortex	0.000			2	8.988E-005	0.004	0.996	0.000
Left motor cortex–Right prefrontal cortex	0.118			2	0.059	2.719	0.081	0.141
Left motor cortex–Right motor cortex	0.031			2	0.016	0.38	0.687	0.022
Right prefrontal cortex–Right motor cortex	0.03			2	0.015	0.455	0.638	0.027
**B. Coefficient**								
	**β**	**Std. Error**			**t**	**Sig.**	**CI 95%**	**Partial Eta Squared**
**Left prefrontal cortex–Left motor cortex: Dependent Variable**								
*Intercept*	1.135	0.179			6.356	0.000	(0.771 to 1.498)	0.550
Responders⁄ Nonresponders to CPM test	-0.516	0.211			-2.447	0.02	(-0.946 to -0.087)	0.154
Responders to CPM test* Multiple psychiatric disorders according to the MINI	-0.006	0.065			-0.097	0.924	(-0.14 to 0.127)	0.000
Nonresponders to CPM test* Multiple psychiatric disorders according to the MINI	-0.222	0.097			-2.275	0.03	(-0.42 to -0.023)	0.136
**Left prefrontal cortex–Right prefrontal cortex: Dependent Variable**								
*Intercept*	1.007	0.206			4.896	0.000	(0.589 to 1.426)	0.421
Responders⁄ Nonresponders to CPM test	-0.302	0.243			-1.243	0.223	(-0.797 to 0.193)	0.045
Responders to CPM test* Multiple psychiatric disorders according to the MINI	0.014	0.075			0.189	0.852	(-0.139 to 0.168)	0.001
Nonresponders to CPM test* Multiple psychiatric disorders according to the MINI	-0.128	0.112			-1.142	0.262	(-0.357 to 0.1)	0.038
**Left prefrontal cortex–Right motor cortex: Dependent Variable**								
*Intercept*	0.619	0.172			3.599	0.001	(0.269 to 0.969)	0.282
Responders⁄ Nonresponders to CPM test	-0.105	0.203			-0.514	0.610	(-0.518 to 0.309)	0.008
Responders to CPM test* Multiple psychiatric disorders according to the MINI	0.004	0.063			0.068	0.946	(-0.124 to 0.133)	0.000
Nonresponders to CPM test* Multiple psychiatric disorders according to the MINI	-0.006	0.094			-0.063	0.950	(-0.197 to 0.185)	0.000
**Left motor cortex–Right prefrontal cortex: Dependent Variable**								
*Intercept*	1.025	0.175			5.838	0.000	(0.667 to 1.382)	0.508
Responders⁄ Nonresponders to CPM test	-0.582	0.207			-2.805	0.008	(-1.004 to -0.16)	0.193
Responders to CPM test* Multiple psychiatric disorders according to the MINI	0.05	0.064			0.775	0.444	(-0.081 to 0.181)	0.018
Nonresponders to CPM test* Multiple psychiatric disorders according to the MINI	-0.211	0.096			-2.2	0.035	(-0.406 to -0.016)	0.128
**Left motor cortex–Right motor cortex: Dependent Variable**								
*Intercept*	0.772	0.243			3.175	0.003	(0.277 to 1.266)	0.234
Responders⁄ Nonresponders to CPM test	-0.334	0.287			-1.163	0.253	(-0.919 to 0.250)	0.039
Responders to CPM test* Multiple psychiatric disorders according to the MINI	0.049	0.089			0.547	0.588	(-0.133 to 0.230)	0.009
Nonresponders to CPM test* Multiple psychiatric disorders according to the MINI	-0.09	0.133			-0.678	0.502	(-0.360 to 0.180)	0.014
**Right prefrontal cortex–Right motor cortex: Dependent Variable**								
*Intercept*	0.558	0.216			2.589	0.014	(0.119 to 0.997)	0.169
Responders⁄ Nonresponders to CPM test	-0.133	0.255			-0.522	0.605	(-0.652 to 0.385)	0.008
Responders to CPM test* Multiple psychiatric disorders according to the MINI	0.072	0.079			0.912	0.369	(-0.089 to 0.233)	0.025
Nonresponders to CPM test* Multiple psychiatric disorders according to the MINI	0.033	0.118			0.281	0.780	(-0.206 to 0.273)	0.002

^*a*^ *R^2^ adjusted = 0*.*198*

^*b*^ *R^2^ adjusted = -0*.*027*

^*c*^ *R^2^ adjusted = 0*.*005*

^*d*^ *R^2^ adjusted = 0*.*192*

^*e*^ *R^2^ adjusted = - 0*.*012*

^*f*^ *R^2^ adjusted = -0*.*019*.

The analysis of the interaction effects revealed as main effect a significant interaction between the CPM-test and the ROI−ROI FC in left MC−left PFC (p = 0.016) and left MC−right PFC (p = 0.018). Multiple psychiatric diagnoses as regressor also presented a significant interaction with nonresponse to the CPM test in these ROI−ROI FC (left MC−left PFC: β = -0.222, t = -2.275, p = 0.03; left MC−right PFC: β = -0.211, t = -2.2, p = 0.035). The interaction in left MC−left PFC between response to CPM-test and FC remained significative despite the presence of psychiatric disorders (adjusted R^2^ = 0.198, β coefficient for the CPM-test = -0.516, t = -2.447, p = 0.02) and in left MC−right PFC (adjusted R^2^ = 0.192, standard β coefficient for the CPM-test = -0.582, t = -2.805, p = 0.008).

## 4. Discussion

These results indicated that nonresponders had an increased ROI−ROI FC between left MC and bilateral PFC. The interaction analysis with the psychiatric disorders revealed that the left MC−bilateral PFC FC was higher in CPM test nonresponders despite psychiatric diagnoses. Nevertheless, psychiatric disorders were associated with increased FC between the left MC and bilateral PFC in the nonresponder group. Together, these results suggest that the increased FC between the left MC and the bilateral PFC in FM is a neural correlate linked to the dysfunction of the DPMS.

The results of this study hint at the distinct function of the right and left PFC on pain processing in FM. The ROI−ROI FC assessed using fNIRS suggests that the strength of the FC between the left MC and the bilateral PFC is differentially present between CPM test responders and nonresponders when coexisting with more than one psychiatric disorder diagnosis. This differential pattern of FC patterns between nonresponders and responders in the current sample of women with FM is interesting, considering that the primary motor cortex (M1) and the DLPFC are target sites for transcranial noninvasive brain stimulation (NIBS) [69]. Additionally, targeting the left MC with NIBS has been shown to reduce pain scores, improving the dysfunction of the DPMS [70]. In the interpretation of the present findings, it is crucial to realize that all patients were right-handed, which is a factor that may be related to the distinct role of brain hemispheres in pain processing.

These results suggest that there is an interaction effect between the mapping of FC and the presence of psychiatric disorders. The most prevalent psychiatric disorder in the sample was major depressive disorder (MDD). The severity of depressive symptoms has already been identified as an important factor for CPM inefficiency [20]. FM and MDD present overlapping features, and patients with FM who have comorbid depression symptoms present more CPM dysfunction than those without them [71]; therefore, it is not entirely surprising that the diagnoses of psychiatric disorders affect FC in patients with FM. Nevertheless, the two nosological entities are distinguishable with the CPM test, with the deficit of pain inhibition being more specific to FM and allowing the differentiation of FM from MDD [27]. In the present study, the coexistence of psychiatric disorder diagnoses displayed a significant interaction effect with the FC between left MC–bilateral PFC in nonresponders (i.e., the group with higher DPMS dysfunction). Nonresponders differed from responders specifically in the FC between left MC–bilateral PFC. Therefore, these results hint at a possible complex relationship between CPM inefficiency and psychiatric disorders in FM.

The association between the coexistence of psychiatric disorder diagnoses and FC in nonresponders — a subgroup of patients that theoretically has poorer DPMS function than responders — may reflect a dysfunctional state underpinning central sensitization’s pathophysiology. This hypothesis finds some plausibility in the concept that FM is a primary pain condition that has as an intrinsic characteristic an important emotional component [72]. The results provide insight that insufficient descending inhibition signaling is partially associated with psychiatric disorders. Although these findings help advance the comprehension of dysfunction processes related to FM, parsimony is required to interpret this interrelationship because our results are correlational and do not allow a causal relationship to be established.

Because the FC between the left MC and the bilateral PFC is associated with response to the CPM test, this seems to reflect the DPMS dysfunction that distinguishes subgroups of patients with FM [20]. According to an earlier study, patients with FM evaluated with transcranial magnetic stimulation (TMS) showed a greater short intracortical inhibition in the left M1 of CPM test nonresponders, which was associated with DPMS dysfunction [23]. Thus, the current results present a potential translational value for NIBS’ use for neurofunctional imaging parameters. This is a promising association for therapeutic development for pain and neurological and psychiatric disorders using NIBS, which is a growing therapeutic tool. Thus, a better understanding of how different treatment parameters interact to produce clinical outcomes is critical to optimize the effectiveness of these new treatments. Unlike medications that only involve the parameter space of choosing whether to use the drug or not, at which dosage, and for how long, neuromodulation devices such as tDCS and repetitive TMS (rTMS) require the additional decisions of the location, frequency, and length of stimulation and whether to stimulate with the anode or cathode.

The present findings suggest the presence of a distinct hyperconnectivity in the target areas involved in pain processing and those used as therapeutic targets to improve pain measures, namely the PFC and MC. The increased FC in the nonresponder group may indicate a disruption of the mechanism mediated by inhibitory gamma-aminobutyric acid (GABAergic) on M1 interneurons, as indexed by short intracortical inhibition previously demonstrated in another study [73]. Simultaneously, it may be the result of an up-regulatory phenomenon of intracortical inhibitory networks, possibly mediated by GABA receptors, which is a biologically plausible hypothesis according to an earlier study that found an association between increased transient short intracortical inhibition and DPMS deficiency [23]. Given that cortical layers are constituted by dense interconnections of pyramidal neurons and inhibitory interneurons, it is impossible to determine which information is being outputted by presynaptic neuronal activity by estimation of the hemodynamic response alone [74–77]. Hence, the higher activity of a particular ROI implicates higher presynaptic neural activity independent of its activity quality (e.g., excitatory or inhibitory synaptic signaling). The current results build on the knowledge of the increased connection between the MC and PFC’s neural networks associated with DPMS dysfunction, which may be a compensatory mechanism to counter-regulate the sustained GABAergic intracortical excitability of chronic pain. The concept of a physiological adaptation response following a prolonged system demand supports this hypothesis and may explain the dysfunctional remapping of related system components, which has been observed in other contexts in autonomic, metabolic, and inflammatory systems [78].

Previous studies have demonstrated that the left and right DLPFC were differentially affected by high frequencies with rTMS or anodal tDCS and that lateralization effects are not negligible [79, 80]. By contrast, low-frequency rTMS has been used to treat depression when the rTMS is applied to the right DLPFC. Furthermore, DLPFC inhibitory effects on the ipsilateral M1 were higher in the left hemisphere than in the right hemisphere [81], even though the right DLPFC has inhibitory effects on the left M1 [82]. Right-handers presented stronger effects from the left-to-right MC, which seems to involve excitatory and inhibitory inter-hemispheric activity [83], supporting the idea of increased activity tone in the left M1 of right-handers. Lastly, FC changes are associated with higher opioid use in left MC–right MC. Although such an association does not prove direct causality, it is compatible with a causal model in which higher doses of opioids may cause cumulative anatomical–functional changes in pain pathways. In contrast to opioids, the nonopioid analgesic used was inversely correlated with connectivity.

This study has several limitations. First, the study design prevents the establishment of a causal nexus, requiring future longitudinal studies comparing patients within groups over time for dynamic characterization of FC patterns and clinical manifestations in FM. In this sense, a series of assessments of both FC and the CPM test could evaluate the reliability and dynamic evolution of response to the CPM-test. In this study, caution is required when interpreting results because the assessments reflect a single measurement using a psychophysical test. Second, this study used a dichotomized approach to classify patients according to their CPM test score. Although categorization into many groups is biologically plausible, our sample was small for using this approach. Third, the sample included only right-handed adult females. Although this profile represents most patients with FM, this factor implies that the current results are not generalizable to the entire FM population. Fourth, the groups in this study’s sample were not equal. Although this imbalance between the groups may reduce the power of analysis, the current findings support key data in research related to an integrative view between the cortical processing of pain and DPMS function. Fourth, the 10–10 system was used for optode positioning. Although the aim was to cover the DLPFC, it was preferred to refer to this ROI generically as the PFC. fNIRS presents superior temporal resolution than fMRI at the expense of lower spatial resolution. In addition to the absence of a neuronavigation system, it was not unequivocally precise to affirm that the activation occurred in a specific area, such as the DLPFC or the M1. Finally, a reduced montage was used to cover cortical sites involved in the top-down references related to the DPMS. As future directions, it is crucial to test whether FC within these FM subgroups is subject to changes after neuromodulation with transcranial direct current stimulation. Because patients with FM who are nonresponders to the CPM test have a poorer response to treatment, it is worth investigating if baseline FC may be a predictor of different outcomes in these patients.

In conclusion, these results provide insights into the functional mapping between critical cortical areas involved in pain modulation. These results reveal the existence of FC differences between pain phenotypes of patients with FM defined by a classical psychophysical test. They indicate that the increased connectivity between the left motor and the prefrontal cortex may be an objective marker of DPMS dysfunction and subject to the interplay between fibromyalgia and psychiatric disorders.

## Supporting information

S1 Table Supporting data. Underlying individual data with the corresponding captions on the file.
(PDF)
