# Supplementary material for: Hyper-connectivity between the left motor cortex and prefrontal cortex is associated with the severity of dysfunction of the descending pain modulatory system in fibromyalgia
Source: PLoS One. 2022 May 27;17(5):e0247629. doi: 10.1371/journal.pone.0247629 (PMC9140239; doi:10.1371/journal.pone.0247629)
Supplement: S1 Table — Underlying individual data with the corresponding captions on the file. (PDF) [file pone.0247629.s001.pdf]

subject	cpm_cat	psychiatric_diagnoses	opioid	n_opioid	Lfc_Lmc	Lfc_Rfc	Lfc_Rmc	Lmc_Rfc	Lmc_Rmc	Rfc_Rmc
1	2.00	2.00	2.00	2.00	.679297	.788206	.548976	.761810	.819433	.864691
2	1.00	1.00	2.00	1.00	.537581	.403188	.334718	.312130	.287628	.266339
3	2.00	2.00	2.00	2.00	.728465	.746816	.511170	.601925	.477118	.526169
4	1.00	2.00	2.00	2.00	.745169	.617113	.688711	.705984	.970521	.741474
5	1.00	2.00	2.00	2.00	.596546	.824981	.536934	.576740	.530685	.624293
6	1.00	2.00	2.00	2.00	.577015	.513591	.462220	.546491	.637490	.533566
7	1.00	2.00	2.00	2.00	.555332	.703584	.512414	.500046	.445460	.449544
8	2.00	2.00	2.00	1.00	.836914	.847282	.806435	.676151	.649847	.701168
9	1.00	2.00	2.00	2.00	.603714	.727585	.518398	.564125	.473563	.517611
10	2.00	2.00	2.00	2.00	.577423	.678094	.670350	.495302	.561129	.792567
11	1.00	1.00	1.00	2.00	.224456	.458652	.230374	.203522	.184336	.251455
12	2.00	2.00	2.00	1.00	.754164	1.009965	.590486	.644501	.491017	.622552
13	2.00	1.00	1.00	1.00	.916237	.958442	.691833	.932100	.766452	.716621
14	2.00	2.00	2.00	2.00	.553416	.510135	.538336	.479978	.437818	.335603
15	1.00	2.00	1.00	2.00	.673689	1.041676	.419703	.567604	.318452	.373579
16	2.00	2.00	2.00	2.00	.817950	.645324	.704493	.570734	.729624	.614158
17	1.00	2.00	2.00	2.00	.574866	.791112	.467346	.522764	.607789	.484159
18	1.00	1.00	2.00	2.00	.754504	.635664	.513341	.484433	.644890	.394074
19	1.00	1.00	2.00	2.00	.491249	.464430	.493531	.333086	.292573	.582870
20	2.00	1.00	2.00	2.00	.942994	.890081	.532384	.762032	.518098	.507763
21	1.00	2.00	2.00	1.00	.689263	.685100	.595992	.497682	.502714	.913120
22	2.00	2.00	2.00	2.00	.788981	.856219	.605795	.709508	.716789	.609613
23	1.00	2.00	2.00	2.00	.438943	.642170	.309090	.511205	.593569	.333256
24	2.00	2.00	2.00	2.00	.569370	.813064	.612728	.549618	.464418	.691381
25	1.00	1.00	2.00	2.00	.582612	.989030	.591185	.577868	.398957	.624576
26	1.00	2.00	1.00	2.00	.360867	.585675	.398113	.279458	.239229	.342640
27	2.00	1.00	2.00	1.00	.880007	.788514	.614374	.747435	.760382	.549151
28	2.00	2.00	1.00	2.00	.773730	.672158	.582626	.682500	.775597	.604789
29	1.00	2.00	2.00	2.00	.674565	.983573	.540321	.606208	.425576	.539536
30	1.00	2.00	2.00	2.00	.717296	.887889	.795345	.657075	.651854	.782867
31	1.00	2.00	2.00	1.00	.737898	.670199	.619878	.509326	.619928	.704875
32	2.00	2.00	2.00	2.00	.526411	.691838	.504662	.462971	.385974	.504590
33	1.00	2.00	2.00	2.00	.707827	.754292	.585454	.733586	.689201	.769995
34	1.00	1.00	2.00	2.00	1.016093	1.046646	.875868	.929999	.979919	.875035
35	1.00	2.00	1.00	1.00	.431313	.573025	.392844	.355267	.319837	.426308
36	1.00	1.00	1.00	1.00	.798960	.862867	.800299	.711008	.855850	.725256
37	1.00	1.00	2.00	2.00	.490066	.893159	.309116	.386918	.246300	.256641

subject	Subject number.
cpm_cat	Conditioned Pain Modulation (CPM) test category:<br>1: Responder;<br>2: Non-responder;
psychiatric_diagnoses	>1 psychiatric disorder according to the MINI:<br>1: Yes;<br>2: No;
opioid	Opioid use per day ≥5 mg by mean morphine-equivalent dose:<br>1: Regular opioid use;<br>2: No use/Minimal opioid use;
n_opioid	Number of days of non-opioid analgesic use in the last month ≥ 4 days per week:<br>1: Yes;<br>2: No;
Lfc_Lmc	Functional Connectivity Z Values between:<br>Left Prefrontal Cortex and Left Motor Cortex;
Lfc_Rfc	Functional Connectivity Z Values between:<br>Left Prefrontal Cortex and Right Prefrontal Cortex;
Lfc_Rmc	Functional Connectivity Z Values between:<br>Left Prefrontal Cortex and Right Motor Cortex;
Lmc_Rfc	Functional Connectivity Z Values between:<br>Left Motor Cortex and Right Prefrontal Cortex;
Lmc_Rmc	Functional Connectivity Z Values between:<br>Left Motor Cortex and Right Motor Cortex;
Rfc_Rmc	Functional Connectivity Z Values between:<br>Right Prefrontal Cortex and Right Motor Cortex;
